# Recent Advances in Engineered Stem Cell-Derived Cell Sheets for Tissue Regeneration

**DOI:** 10.3390/polym11020209

**Published:** 2019-01-26

**Authors:** Hyunbum Kim, Yunhye Kim, Jihyun Park, Nathaniel S. Hwang, Yun Kyung Lee, Yongsung Hwang

**Affiliations:** 1 Soonchunhyang Institute of Medi-bio Science (SIMS), Soonchunhyang University, Cheonan-si, Chungcheongnam-do 31151, Korea; tiggerhy@sch.ac.kr (H.K.); kyhye4@naver.com (Y.K.); purjur311@naver.com (J.P.); 2 School of Chemical and Biological Engineering, the Institute of Chemical Processes, Seoul National University, Seoul 08826, Korea; 3 The BioMax Institute of Seoul National University, Seoul 08826, Korea

**Keywords:** cell sheet engineering, smart polymer, temperature-responsiveness, tissue regeneration, stem cell, scaffold-free

## Abstract

The substantial progress made in the field of stem cell-based therapy has shown its significant potential applications for the regeneration of defective tissues and organs. Although previous studies have yielded promising results, several limitations remain and should be overcome for translating stem cell-based therapies to clinics. As a possible solution to current bottlenecks, cell sheet engineering (CSE) is an efficient scaffold-free method for harvesting intact cell sheets without the use of proteolytic enzymes, and may be able to accelerate the adoption of stem cell-based treatments for damaged tissues and organs regeneration. CSE uses a temperature-responsive polymer-immobilized surface to form unique, scaffold-free cell sheets composed of one or more cell layers maintained with important intercellular junctions, cell-secreted extracellular matrices, and other important cell surface proteins, which can be achieved by changing the surrounding temperature. These three-dimensional cell sheet-based tissues can be designed for use in clinical applications to target-specific tissue regeneration. This review will highlight the principles, progress, and clinical relevance of current approaches in the cell sheet-based technology, focusing on stem cell-based therapies for bone, periodontal, skin, and vascularized muscles.

## 1. Introduction

Stem cell-based therapies have significant therapeutic potential and present substantial benefits over conventional treatment strategies in various diseases [1]. Consequently, many studies have focused on the development of stem cell-based therapies and investigating their therapeutic potential for the treatment of devastating diseases [2]. Among various stem cell types reported so far, human pluripotent stem cells (hPSCs), including human embryonic stem cells (hESCs) and human induced pluripotent stem cells (hiPSCs), possess the most robust capabilities for self-renewal and differentiation into one or more specialized cell types for regenerating damaged tissue [3]. However, the clinical application of hPSCs has been hampered by several factors. These include safety concerns such as immunological rejection and teratoma formation, as well as ethical issues, which have limited the clinical application of hESCs [4]. Although hiPSCs can be derived from autologous patient-specific resources, and therefore could bypass most immunological and ethical concerns. However, their incomplete fate determination into target-specific cells upon in vivo transplantation may cause teratogenic risks due to their pluripotency and self-renewable nature, a problem which remains unsolved [5]. Because human mesenchymal stem cells (MSCs) can be derived from a patient’s own tissue, including their bone marrow, adipose tissue, cord blood, and tonsils, and show reasonable regenerative potential [6,7,8], these cells are the most favored for clinical applications [9].

The ultimate goal of stem cell-based therapies is to promote the regeneration of tissues/organs damaged by disease, injury, trauma, or aging-associated degenerative disorders [10]. Effective clinical application of stem cell-based therapies is dependent not only on the cell types themselves but also on the transplantation procedures [11]. The efficiency of the initial transplantation is determined by the number of trial sites and the cell processing techniques [12,13]. It has been shown that enzymatic treatments for detaching cells prior to in vivo transplantation affect transplanted cell functions within the host tissues—including localization difficulties and poor survival rates, owing to alterations in cell niches and surface proteins [14].

Therefore, to achieve higher efficacy and functionality of stem cell-based therapeutics, numerous studies introduced synthetic polymer-based hydrogels as cell scaffolds, including poly(ethylene glycol) (PEG), poly(vinyl alcohol) (PVA), poly(acrylic acid) (PAA), poly(2-hydroxyethyl methacrylate) (PHEMA), poly(lactic acid) (PLA), poly(glycolic acid) (PGA), and poly(caprolactone) (PCL) for modulating the adhesion, migration, differentiation, and proliferation of stem cells [15,16,17]. These hydrogels are soft materials composed of cross-linked three-dimensional (3D) polymer networks, and have been extensively used over the years to provide cells with 3D structural and instructive cues, owing to their structural similarity to the native extracellular matrix [18,19]. Many studies have incorporated passive and dynamic biochemical cues onto polymer chains in the hydrogel to grant them specific biological functions [20,21].

As an alternative to scaffold-based methods, scaffold-free tissue engineering approaches have been proposed and pioneered by the Okano research group [22,23]. These approaches involve cultivation of cells in temperature-responsive polymer-immobilized tissue culture polystyrene (TCPS) that is fabricated by co-grafting [24,25] and vapor-phase deposition of plasma-polymerized poly(*N*-isopropylacrylamide) (PNIPAAm) [26,27]. The temperature-responsive properties of PNIPAAm-grafted onto TCPS enable rapid detachment of cell sheets without enzymatic treatment [28,29]. In their previous studies, this group used a thermo-responsive polymer, PNIPAAm—detailed principles and applications of which are described in Section 2 and Section 3—that shows reversible volumetric changes in response to surrounding temperatures [28,30]. Methylcellulose (MC) is one of the most extensively investigated temperature responsible polymers, which is derived from cellulose by substituting hydrophilic hydroxyl groups with hydrophobic methoxy groups, and therefore, displays sol-gel transition upon changes in the surrounding temperature. [31,32]. Numerous studies fully demonstrated that cell sheets harvested from the PNIPAAm-/MC-based smart surfaces maintained their intrinsic physiological functions, including intact cell–cell junctions and cell-secreted extracellular matrices (ECMs), while cells detached by conventional mechanical or enzymatic treatments showed both inferior cell phenotypes and the loss of cell-secreted ECMs [33,34]. The same results were observed in a variety of cell types, including epidermal keratinocytes [35], vascular endothelial cells [36], corneal epithelial cells [37], and cardiomyocytes [38,39]. These pioneering studies initiated a new era of scaffold-free tissue engineering, or “cell sheet engineering (CSE)” [23].

**Figure 1:**
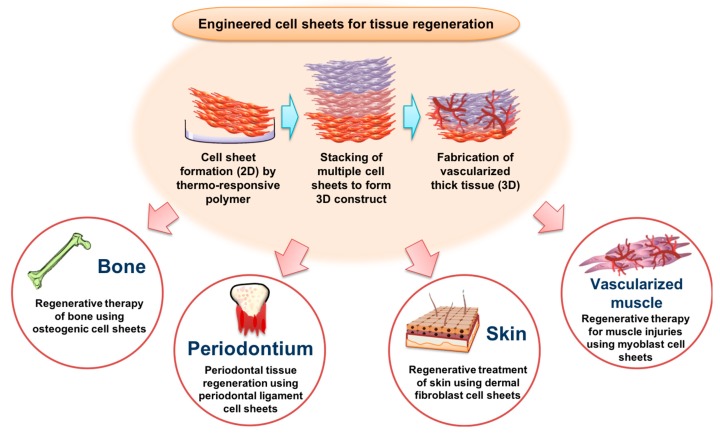
Schematic illustrations of temperature-responsive three dimensional (3D) vascularized cell sheet constructs for tissue regeneration.

In this review, we highlight recent advancements in the creation of functional cell sheets for engineered bone, periodontal, skin, and vascularized muscle (summarized in Figure 1).

## 2. The Principles of Temperature-Responsive Behaviors of Polymers in Terms of Their Critical Solution Temperature

In the past decade, numerous attempts to control the physical properties of polymer-based hydrogels by controlling environmental stimuli, such as pH, temperature, electrical field, magnetic field, and light, have been made for a wide range of biological applications [40,41]. Of these, temperature is the most common environmental stimulus and is easily controllable within the ambient-to-physiological temperature ranges. Hydrogels incorporating temperature-responsive polymers exhibit reversible volume phase transitions (VPT) at defined temperatures [42,43]. The solubility and hydrophilicity of these polymers in aqueous solution is determined by their critical solution temperatures, and allows temperature-responsive polymers to be categorized into two types: those having a lower critical solution temperature (LCST) such as poly(*N*-isopropylacrylamide), poly(*N*,*N*-diethylacrylamide) (PDEA), poly(methyl vinyl ether) (PMVE), poly(*N*-vinylcaprolactam) (PNVCL), and methylcellulose (MC); and those having an upper critical solution temperature (UCST) including PAA, PVA, and PHEMA [44,45]. Polymers with an LCST become insoluble (phase separation of insoluble polymers from the solvent) at temperatures above their LCST, while those with a UCST become soluble at temperatures above their UCST. It is generally believed that LCST and UCST behaviors are mainly determined by the molecular competitions between hydrophilic and hydrophobic moieties within the polymer chains over water molecules upon temperature change [46].

**Figure 2:**
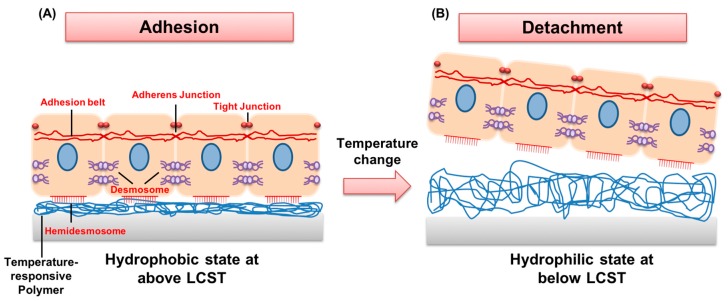
A temperature-responsive matrix showing the attachment and detachment of cell sheets while preserving cell–cell junctions. (**A**) At 37 °C, the cells attach to the surface that is hydrophobic (above lower critical solution temperature). Cells connect to each other by various cell-to-cell junctions and deposit ECMs. (**B**) At values below LCST, cells detach from the hydrophilic surface. Temperature-responsive culture surfaces are able to preserve the pre-existing cell-to-cell junctions and cell-secreted extracellular matrices (ECMs).

The most widely studied temperature-sensitive polymer thus far is PNIPAAm. This polymer undergoes a discontinuous and reversible phase transition, and has an LCST of 32 °C in aqueous solutions [47,48]. For example, at temperatures above the LCST, increased intra- and inter-molecular hydrophobic interactions among isopropyl groups of PNIPAAm result in the release of water molecules through the hydrated coil-to-collapsed globule transition (Figure 2). This enables cell adherence and proliferation on a PNIPAAm-immobilized matrix. In contrast, below its LCST, hydrogen bonding between water molecules and hydrophilic moieties (amide groups) of PNIPAAm dominates, which leads to rapid detachment of cell sheets with a variety of intact cell–cell junctions and cell-secreted extracellular matrices (ECMs) [49].

Since the temperature sensitivity of PNIPAAm-based hydrogels and their capacity to undergo volumetric change are dependent on their chemical and physical structures—mainly the hydrophilic–hydrophobic balance among its side-chains—many studies have tailored the thermal behaviors (LCST) of these hydrogels to be close to physiological temperature, through the incorporation of hydrophilic co-monomers such as poly(methacrylic acid) (PMAA) and poly(ethylene oxide) (PEO), or to be lower than that of PNIPAAm itself by introducing hydrophobic co-monomers such as *n*-butyl methacrylate (*n*-BMA), poly(propylene oxide) (PPO), and poly(ethyl acrylate)(PEA) [50,51,52,53,54].

Similarly, MC exhibits a reversible sol–gel transition in aqueous solution at its LCST of 60 °C [55]. When surrounding temperature increases above its LCST, the MC solution undergoes thermo-reversible gelation due to the hydrophobic interactions between the methyl groups. In addition, when the surrounding temperature decreases below its LCST, gelated MC hydrogel rapidly becomes a solution, making MC an excellent alternative for PNIPAAm to achieve temperature-sensitive matrix-mediated cell sheet formation. Furthermore, the LCST of MC is easily tunable by multiple factors, such as the degree of substitution of methoxy groups, salt concentrations in aqueous solution, the concentration of MC, and the molecular weight of MC [56,57,58].

Another interesting approach and an alternative to PNIPAAm and MC is the development of temperature-sensitive polymers which include the use of 2-(2-methoxyethoxy) ethyl methacrylate (MEO_2_MA) and oligo(ethylene glycol) methacrylate (OEGMA), which display LCST values of 26 and 90 °C, respectively [59]. In this study, the final LCST of MEO_2_MA-*co*-OEGMA hydrogels was precisely controlled by altering the length of the hydrophilic side chain, ethylene oxide (EO), of OEGMA or the molecular ratios between MEO_2_MA and OEGMA. Furthermore, OEGMA possesses non-cytotoxic and anti-immunogenic properties, and has a more uniform thermal profile than PNIPAAm, Lutz and coworkers developed co-polymers of MEO_2_MA and OEGMA which possess LCST close to physiological temperatures [60].

Taken together, these results suggest that the thermal properties of temperature-sensitive polymers can be precisely regulated and therefore can provide promising features for biomedical applications.

## 3. Potential Biomedical Applications of Temperature-Responsive Polymers in Regenerative Medicine and Tissues Engineering

### 3.1. Engineered Bone Tissue Regeneration by CSE

**Figure 3:**
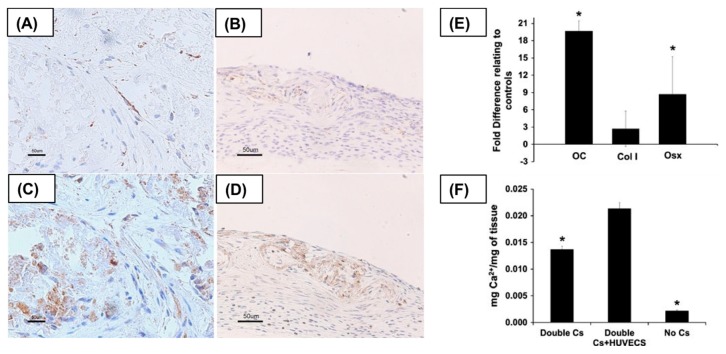
Enhancement of bone-forming ability by co-cultured rat bone marrow stromal cells (rBMSCs) and human umbilical vein endothelial cell (HUVEC)-derived cell sheets (CSs) generated by PNIPAAm-grafted TCPS. Deposition of bone minerals within host tissues after the co-cultured CS implantation. (**A**,**C**): Co-cultured CS. (**B**,**D**): Monoculture of rBMSC CSs. Immunostaining of SRY (**A**,**B**) and osterix (**C**,**D**). The increase in osteogenic matrix in the dorsal flap in mice after implantation of co-cultured CS. (**E**,**F**) Reproduced with permission from [64].

Reconstruction of critical bone defects remains a particularly significant challenge in orthopedic surgery. The current gold standards for addressing these issues involve the use of autologous bone grafts [61], allografts [62], and cell-free bone substitute materials, such as metal alloys or inorganic ceramics [63]. Of the various approaches available, CSE has shown its potential as an alternative therapeutic measure. The engineered osteogenic cells have regenerative capacity and can improve in vitro osteogenic differentiation of MSCs, and in vivo functional bone formation as shown in Figure 3 [64,65].

In earlier studies, Akahane and his colleagues developed a method to achieve osteogenically differentiated cell sheets by lifting cell sheets using a scraper [66,67]. In their studies, prior to in vivo cell transplantation, human bone marrow-derived mesenchymal stem cells (hBM-MSCs) were initially induced to differentiate into the osteogenic lineage in vitro. These cells, without any scaffolding material, were then harvested and transplanted into subcutaneous sites in rats. Their histological analyses revealed that transplanted cell sheets were able to form bone matrices in vivo four weeks post-transplantation.

In another study, Kira et al. developed tricalciumphosphate (TCP) bone grafts wrapped with CSE-based osteogenic cell sheets in order to promote in vivo bone formation in athymic rats and sheep models [68]. They harvested bone marrow cells from sheep, induced them to form osteogenically pre-committed osteogenic cell sheets by detaching the sheets using a scraper, and wrapping the TCP bone grafts with the detached cell sheets. The results demonstrated that these osteogenic cell sheets-wrapped TCP bone grafts successfully promoted in vivo bone regeneration in large bone defect models. Significantly higher alkaline phosphatase activity and robust deposition of both organic and inorganic bone matrices were observed in newly regenerated bone tissues.

Given the great potential of CSE-based approaches developed without using temperature-responsive cell culture substrates, Pirraco et al. developed CSE-based osteogenic cell sheets by culturing bone marrow stromal cells in PNIPAAm-immobilized TCPS [69]. In this study, they achieved osteogenic cell sheets containing cell-secreted dense bony matrices upon in vitro osteogenic commitment, and evaluated their in vivo osteogenic potential for reconstructing in vivo bone formation. These osteogenic cell sheets were subcutaneously transplanted into mice and their in vivo osteogenic potential was histologically analyzed. Newly formed bone matrices were observed seven days post-transplantation, and newly vascularized marrow was found after six weeks, which clearly demonstrate the osteogenic potential of the cell sheets both in vitro and in vivo.

Similarly, Shotorbani et al. used a temperature-responsive poly(*N*-isopropyl acryl amide-*co*-methacrylic acid) (p(NIPAAm-*co*-MMA)) matrix to fabricate ASC-derived osteogenic cell sheets [70]. In this study, they evaluated the effect of two important factors—the secretome of ASCs (SE) and vitamin C (VC)—on cell sheet formation and in vitro osteogenesis of ASC-derived cell sheets. Their p(NIPAAm-*co*-MMA) matrix promoted rapid detachment of osteogenically committed cell sheets within 6 min without any detrimental effects. Furthermore, the combinatorial use of SE and VC improved osteogenic ECM-rich cell sheet formation and prevented senescence of ASC-derived osteogenic cell sheets.

Similar approaches have been made to achieve cell sheets using a thermo-reversible MC-based matrix. For example, Forghni et al. reported a comparative study to evaluate whether PNIPAAm- and MC-coated TCPS dishes were able to support in vitro osteogenic differentiation of human adipose-derived stem cells (hASCs) [71]. Their results demonstrated that both PNIPAAm- and MC-coated TCPS dishes were able to support the detachment of hASCs in the form of cell sheets by lowering the surrounding temperature to 25 °C. In particular, at 25 °C, which is below the LCST of MC, MC was solubilized and led to the detachment of hASC sheets. The detached cell sheets from both PNIPAAm- and MC-coated TCPS were re-attached into collagen-coated TCPS, and these hASC-derived cell sheets were further induced to differentiate into the osteogenic lineage for 21 days, and their phenotypes such as cell shape, viability, growth, and in vitro osteogenic potential were characterized. The in vitro cytotoxicity results revealed that cell viability of hASC-derived cell sheets generated from both PNIPAAm- and MC-coated TCPS dishes were highly viable. Furthermore, these hASC-derived cell sheets were able to undergo in vitro osteogenic differentiation, which was confirmed by positive staining of alkaline phosphatase and Alizarin Red as well as up-regulation of osteogenic marker genes, such as osteocalcin.

In combination, these diverse studies suggest that the cell sheet-mediated regenerative potential of bone grafts is very promising and has applications in clinical studies.

### 3.2. Engineered Periodontal Tissue Regeneration by CSE

Periodontitis is a common set of inflammatory diseases that induce the destruction of tooth-supporting tissues, including the progressive loss of the alveolar bone, cementum, and periodontal ligament (PDL) tissues, which eventually result in the loosening and subsequent loss of teeth [72]. Although clinically available treatments such as scaling, guided tissue regeneration, and surgical cleaning are employed for the restoration of periodontal tissue [73,74,75], the extent to which these treatments can remove necrotic tissues and regulate inflammation is limited, and treatment can result in weak attachment of newly generated tissue and only partial restoration of periodontal tissue [76,77,78]. Thus, complete regeneration in serious pathological conditions by the aforementioned methods is seldom achieved and is often associated with poor clinical outcome [77].

**Figure 4:**
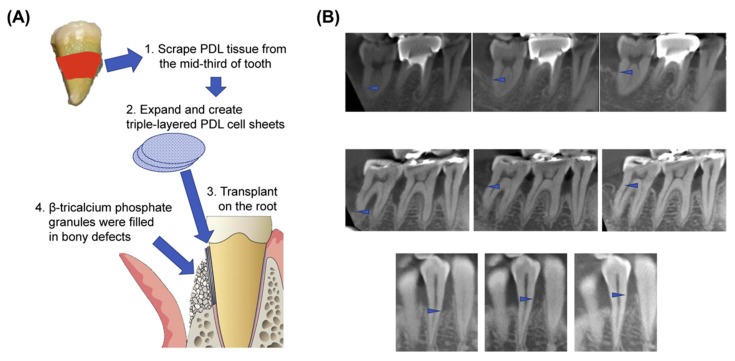
Periodontal tissue engineering with autologous periodontal ligament (PDL)-derived cell sheets. (**A**) Schematic diagram of the implantation of multi-layered patient-derived PDL cell sheets induced by PNIPAAm-immobilized TCPS. (**B**) Regeneration of periodontal tissues with increased bone height in representative cases. Arrowheads: the most apical part of bone defects. Reproduced with permission from [82].

To overcome these challenges, recent approaches have involved the use of stem cell- and periodontal ligament cell-based treatments in conjunctions with CSE for periodontal regeneration [79,80,81]. It has been speculated that PDL cell sheets, acquired from periodontal ligament-derived cells grown on PNIPAAm-immobilized TCPS, mimic the growth pattern of PDL and manipulate cellular signals that improve the regeneration of periodontal tissues as shown in Figure 4 [82]. Hasegawa et al. engineered human PDL cell sheets using PNIPAAm-immobilized TCPS and evaluated their in vivo engraftment efficiency [83]. Their results demonstrated that human PDL cell sheets transplanted into an athymic rat model of mesial dehiscence contributed to the regeneration of PDL tissues, having a large number of fibrils anchored to an acellular cementum-like layer. This recapitulated the structures and regeneration processes of native PDL fibers.

For instance, Tsumanuma et al. compared the regenerative potential of PDL cell sheets consisting of a single cell type derived from different mesenchymal tissues, such as periodontal ligament (PDL), alveolar periosteum (AP), and iliac bone marrow (BM) in a canine one-wall intrabony defect model [84]. They fabricated three-layered cell sheets using each cell source with a supporting polyglycolic acid (PGA) membrane for cell sheets, which is an FDA-approved biomaterial for clinical use, and transplanted these cell sheets into the denuded root surface. Their results revealed that only PDL-derived stem cells promoted PDL regeneration eight weeks post-transplantation, which was evident by the newly formed cementum and well-oriented PDL fibers, suggesting that PDL-derived stem cells would be more suitable to treat periodontitis than other stem cells.

Similarly, to evaluate the potential of PDL cell sheets for use in clinical applications, researchers investigated their efficacy using larger animal models. Iwata et al. fabricated transplantable three-layered canine cell sheets, consisting of osteogenically and cementogenically pre-committed PDL-derived cells with an osteoinductive medium, using PNIPAAm-immobilized TCPS. Furthermore, to facilitate the transfer of cell sheets upon detachment, and to improve the in vivo efficacy of transplanted target cell sheets to the defects, an additional layer consisting of a polyglycolic acid (PGA) membrane was introduced as physical support for cell sheets. These cell sheets were transplanted into dental roots of canine periodontal defect models [79]. The results clearly demonstrated that PDL-derived cells could undergo both osteogenic and cementogenic differentiation within host tissues, and promote new bone and cementum-like tissue formation at the defect sites.

The results of these studies suggest that transplantation of PDL-derived cell sheets with clinically-relevant protocols represent a possible alternative treatment option for periodontitis patients.

### 3.3. Engineered Skin Regeneration by CSE

In contrast to conventional therapies that cannot reliably improve non-healing wounds or reverse pathological scarring, cell-based therapies show immense promise for the treatment of non-healing wounds [85,86]. Recent work has addressed the problems of loss of plasticity and off-target delivery through the application of modern bioengineering techniques. Because of their integral role in the native wound repair process, human MSCs and adipose-derived stem cells (ASCs) constitute a promising cell source for novel cell-based therapies [87,88]. It is, therefore, necessary to assess the viability of cell-based therapeutics using hMSCs derived from bone marrow, umbilical cord, and adipose tissue for the treatment of non-healing wounds and scars [89,90,91].

**Figure 5:**
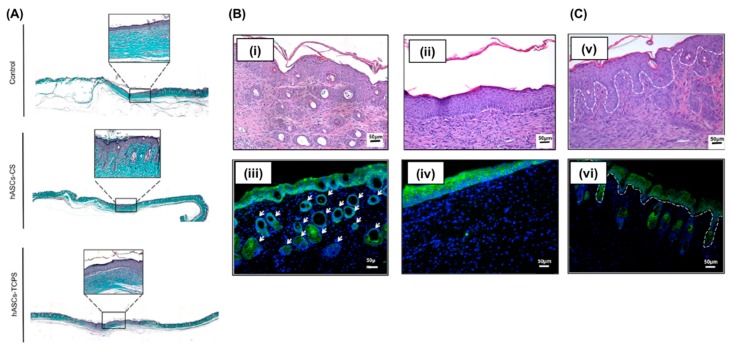
Epidermis regeneration by human adipose-derived MSCs-CS engineering in full-thickness wound skin mice. (**A**) Formation of Rete Ridges-like epidermis in hMASC-CS group (Masson’s trichrome staining). (**Bi,iii**) Hair-follicle (white arrows) formation in neo-epidermis at the hAMSC-CS transplanted defect sites, and (**Bii,iv**) hAMSC-TCP transplanted groups. (**Cv,vi**) Epidermis morphogenesis driven by hAMSCs-CS transplantation after 21 days. Keratin 14 (green), and DAPI (blue); scale bar = 50 μm. Reproduced with permission from [94].

Advancements in stem cell-based therapeutics over several years have led to the development of approaches which employ stem cell-derived cell sheets for wound healing [92]. These cell sheet-based technologies use temperature-responsive polymers to lift the pre-cultured or pre-treated cell sheets while maintaining the cell-deposited skin-associated ECMs, and the technique is remarkably effective for skin regeneration [93]. Cerqueira et al. developed multiple stacked 3D cell sheets derived from hASCs using PNIPAAm-immobilized TCPS and compared their in vivo engraftment and skin regeneration potential to that of transplanted cells generated by enzymatically detached methods using a full-thickness wound model as shown in Figure 5 [94]. Their results showed that hASC-derived cell sheets transplanted into mice with full-thickness excisional wounds could promote neovascularization and extensively influence epidermal morphogenesis through the dynamic interaction between transplanted cell sheets and resident cells, and promotion of paracrine factors. This resulted in the regeneration of thicker epidermis, and the formation of new hair follicles, and could be achieved through a higher degree of cell engraftments via cohesive cell–cell and cell–ECM interactions.

Similarly, Lin et al. generated single- and multi-layered cell sheets composed of hASCs using PNIPAAm-immobilized TCPS, transplanted them directly to the full-thickness wound bed, and assessed their wound healing capacity against untreated controls [95]. Their in vivo experimental results demonstrated that cell-secreted extracellular structures, and the organization of transplanted multi-layered cell sheets, were maintained within the in vivo environment. Histological evaluation showed injury areas were significantly smaller compared to their control counterparts. Moreover, they found that wounds treated with multi-layered cell sheets contained more newly synthesized collagen matrices and displayed slightly enhanced blood vessel structures and density.

Recently, Kato et al. evaluated the efficacy of rat adipose-derived stem cell (rASC) sheets, which were generated by PNIPAAm-immobilized TCPS, as a novel treatment method for diabetic ulcers using a full-thickness skin defect with exposed skull bone in the total absence of periosteum, using type 2 diabetic and obesity models [96]. In this in vivo study, allogenic rASCs were used to fabricate rASC-derived cell sheets which were directly transplanted into the defect sites with artificial skin grafts (PELNAC; Smith & Nephew, Tokyo, Japan). Surprisingly, transplanted rASC-derived cell sheets significantly accelerated the wound closure time and promoted dense dermis tissue formation 14 days post-transplantation. Furthermore, transplanted rASC-derived cell sheets were able to present various angiogenic factors, such as vascular endothelial growth factor (VEGF) and hepatocyte growth factor (HGF) within the in vivo microenvironment, and rASC-derived cell sheets could contribute to the formation of newly vascularized networks by paracrine effects.

Recent studies into the application of CSE in skin regeneration have yielded promising results and suggest that novel strategies utilizing engineered stem cell-derived cell sheets can provide a functional cell source for tissue regeneration with the transplanted cell-secreted release of various growth factors.

### 3.4. Engineered Muscle Regeneration by Pre-Vascularized CSE

Neovascularization within host microenvironments upon transplantation is critical for successful engraftment, sustaining cell sheet-based thick three-dimensional tissue constructs, and the adequate delivery of nutrients and oxygen to the transplanted cells [97]. Several strategies based on transplantation of either the endothelial cells themselves or incorporation of angiogenic factors such as vascular endothelial growth factor (VEGF), were proposed to form new blood vessel-like structures in cell-laden tissue constructs [98,99]. However, most of these strategies were associated with challenges such as a shortage of cell sources, insufficient maturation of the newly formed vessels, and potential tumorigenicity of growth factors [100,101,102]. Therefore, efficient methods to promote neovascularization local to transplanted cells and their surrounding host tissues are necessary to achieve fully functional engraftment of transplanted cell sheets.

In order to achieve the aforementioned pre-vascularization required for functional tissue regeneration, Masumoto et al. developed methods to induce differentiation of human iPSCs into cardiomyocytes, endothelial cells, and vascular mural cells, for developing vascularized cardiovascular cell sheets [103]. Prior to pre-vascularized cell sheet formation, directed differentiation of undifferentiated hiPSCs into the aforementioned lineages was induced for 14 days, after which these pre-committed cells were re-plated into PNIPAAm-grafted TCPS to form pre-vascularized cardiac cell sheets. The results indicated that transplantation of pre-vascularized cardiac cell sheets into the infarcted hearts of rat models significantly improved long-term survival of transplanted cells, and their subsequent engraftment efficiency. This resulted in enhanced cardiac function, with the successful generation of vascularized structures. These findings led to subsequent studies focused on CSE-based regenerative medicine for the treatment of chronic ischemic injury [104]. In this study, Okano and co-workers reported a synergetic effect of transplanting both cardiac stem cell sheets generated by PNIPAAm-immobilized TCPS and endothelial progenitor cells, which dramatically improved cardiac function in ischemic epicardium patients. The combined treatment showed the greatest recovery of the endocardium and capillary density, and reduced cardiac fibrosis in the ischemic area compared to control groups, who received either transplantation of single cell sheets or sham surgery.

Another study proposed an alternative method using the stromal vascular fraction (SVF) of adipose tissues to develop angiogenic cell sheets for the potential generation of complex cell-laden tissue constructs [105]. As cells present in the SVF have been found to generate numerous angiogenic factors under hypoxic conditions, the Reis research group applied these SVFs in cell sheet-based tissue engineering approaches to increase the newly formed vascular networks of transplanted cell sheets, which could be dramatically enhanced under hypoxic conditions [106]. In this study, to improve neovascularization within the host microenvironment without introducing additional growth factors or different cell types, for the first time, they developed SVF-derived cell sheets by detaching sheets using a tweezer, and demonstrated that these cell sheets could secrete angiogenic factors, including VEGF and FGF, under hypoxic conditions, which was evident by immunocytochemistry against CD31 and CD146. Their results clearly indicated that the use of SVF-derived cell sheets was able to improve the restoration of blood flow in a mouse hind-limb ischemia model.

Employing another approach to promote neovascularization, Rayatpisheh et al. introduced electrospinning techniques into cell sheet engineering to create aligned tubular constructs of human aortic smooth muscles cells [107]. In this study, to engineer uniaxially aligned and tubular blood vessel structures, they fabricated micro-patterned and PNIPAAm-grafted polydimethylsiloxane (PDMS) substrates, and then a partial area of these substrates was sandwiched by uniaxially aligned electrospun polycaprolactone (PCL) scaffolds. Once the smooth muscle cells cultured on these substrates became confluent and aligned along with the micropatterns, by lowering the surrounding temperature, the uniaxially aligned smooth muscle cell sheets were detached. Finally, the detached cell sheets were rolled using a Teflon mandrel to achieve tubular structures as an analog to a small diameter blood vessel’s internal elastic lamina. These unidirectionally aligned smooth muscle cell sheets could be induced to form blood vessel-like cellular orientation and to express contractile genes.

Similar approaches have been applied to the development of cell sheets with uniaxially aligned structures. For example, Takahashi et al. demonstrated the potential role of manipulating the cellular alignment within cell sheets in creating three-dimensional anisotropy, which closely mimics the orientation/organization of cell and extracellular matrix proteins in native tissues [108,109]. In their studies, they fabricated a micro-patterned PNIPAAm-immobilized cell culture substrate and revealed the important role of biomimetically-engineered anisotropy for controlling the orientation within scaffold-free three-dimensional skeletal muscle constructs, to mimic complex structures of skeletal muscle tissues.

**Figure 6:**
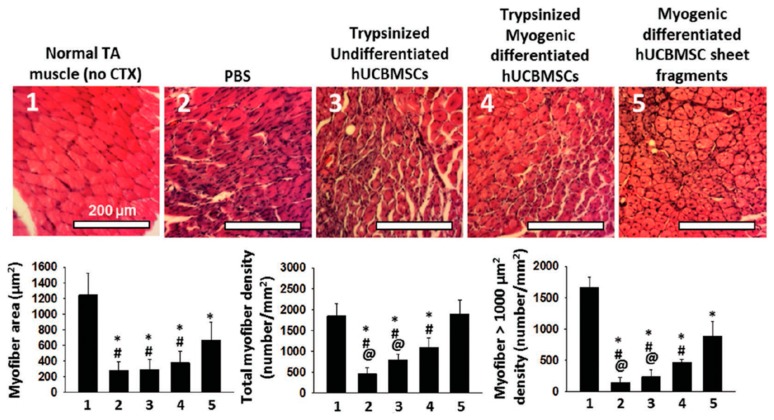
Skeletal muscle regeneration after transplantation of myogenically differentiated human umbilical cord blood mesenchymal stem cell (hUCBMSC) sheet fragments in cardiotoxin (CTX)-induced mice models. hUCBMSC derived myogenic differentiated cell sheets improved tibialis anterior (TA) muscle regeneration estimated by hematoxylin and eosin staining. Restoration of TA muscle specific extracellular matrix (ECM) (laminin) Scale bar = 100 μm. Reproduced with permission from [110].

Recently, Kim and his co-workers created a novel elastic PNIPAAm-based piezoelectric substrate and evaluated its potential application as a biomimetic matrix, for applying both electrical and mechanical stimuli into skeletal muscle cell sheets generated from human umbilical cord blood-derived mesenchymal stem cells (hUCB-MSCs) [110]. They successfully applied cyclic stretching and bending, as well as electrical stimuli, to the hUCB-MSC sheets, finding that these stimulations promoted robust myogenic differentiation of hUCB-MSCs through intracellular calcium-associated signaling. Their in vivo experimental results further validated the in vivo regenerative capacity of hUCB-MSCs-based skeletal muscle cell sheets, showing that these electromechanically stimulated cell sheets could promote cardiotoxin (CTX)-injured tibialis anterior (TA) muscle regeneration as shown in Figure 6.

Another study reported by Shimizu et al. evaluated the potential applicability of CSE for the treatment of heart tissue failure [111]. Neonatal rat cardiomyocytes were harvested as an intact cell sheet using PNIPAAm-grafted TCPS, and stacked into cell-dense multiple layered myocardial cell sheet grafts. Thick myocardium (~1 mm) cell sheet grafts were created by repeated transplantation of multi-layered cell sheets into dorsal subcutaneous sites, and myocardial cell sheet grafts were surgically connected with arteries and veins of host tissues, resulting in a well-organized microvascular network formation within host tissues. These myocardial cell sheet-based grafts were able to beat synchronously in a unidirectional orientation, even after detachment from the cell culture substrate. Moreover, these cell sheets could regain higher pulsatile amplitude after manipulation and re-orientation into a two-dimensional plane. Adding on to this study, Kawamura et al. reported a promising strategy for improving the viability of transplanted cells by combining human iPSC-derived cardiomyocyte sheets with an omental flap [112]. In their study, the enhanced blood supply provided through the omental flap contributed to the increased viability of the transplanted cells.

In addition to the aforementioned PNIPAAm-based thermoreversible cell culture substrates, numerous studies demonstrated the potential use of temperature-responsive MC as a novel method to generate cell sheets for treating myocardial infarction. Sung and his colleagues developed MC-immobilized cell culture substrates to fabricate rat bone marrow-derived MSC (BM-MSC) sheets and evaluated their regenerative potential using rat myocardial infarction models [113]. In this study, when rat BM-MSCs attained confluence, MC hydrogels were solubilized by changing the surrounding temperature to 20 °C, and a stainless screen was placed on top of cell sheets, resulting in the release of slightly dissociated cell sheet fragments. Finally, these rat BM-MSC-derived cell sheet fragments were directly transplanted into the ischemic left ventricular (LV) wall and their engraftment was evaluated by echocardiography. The in vivo results clearly indicated that transplantation of rat BM-MSC sheet fragments could increase vascular density and improve LV functions 12 weeks post-transplantation. Using the same experimental setup, the group further investigated the efficacy of cell sheet fragments derived from human amniotic fluid stem cells (hAFSCs) using rat myocardial infarction models [114]. Similarly, hAFSC-derived cell sheet fragments detached from MC-immobilized TCPS were able to preserve the cell-secreted endogenous ECM and their cell phenotypes, including Vimentin and SSEA-4 expressions, were maintained after cell sheet fragmentation. When these hAFSC-derived cell sheet fragments were transplanted into the ischemic left ventricular (LV) wall, transplanted cell sheet fragments could regulate multiple angiogenic cytokines within host microenvironments, promote in vivo survival and their engraftment, and improve LV wall thickness as well as the infarct size, compared to their counterpart, dissociated hAFSCs.

These studies suggest that pre-vascularization-based cell sheet approaches for both cardiac and skeletal muscle tissue repairs in the clinical application are very promising.

## 4. Recent Clinical Studies of CSE-Based Approaches

In past decades, CSE has emerged as an alternative technology for improving clinical efficacy and preservation of the biological and biochemical stem cells niches, both for treating various diseases and for regeneration of multiple degenerative tissues [115,116]. These clinical approaches have included numerous efforts to establish effective methods for creating bioengineered scaffold-free three-dimensional CSE-based tissues by layering or stacking multiple cell sheets in an advanced, controlled manner [93,117,118].

The Okano research group conducted a clinical trial in which they treated dilated cardiomyopathy using autologous myoblast cell sheets derived from a 56-year-old patient, which were generated using PNIPAAm-immobilized TCPS [119]. In this study, transplantation of the patient’s own myoblast cell sheets remarkably improved the clinical conditions, without any adverse effects such as arrhythmia, and the use of a left ventricular assist system for further assistance. Similarly, Miyagawa et al. utilized CSE to maximize paracrine effects for the recovery of functional cardiac muscle in ischemic cardiomyopathy patients [120]. In their study, they utilized PNIPAAm-grafted TCPS to acquire autologous somatic tissue-derived cell sheets, which were transplanted over the LV free wall. In patients who received the transplants, remarkable improvements including a reduction in pulmonary pressure, pulmonary vein resistance, and LV wall stress were observed. Another clinical trial of PNIPAAm-grafted surface-mediated multi-layered skeletal muscle-derived cell sheet transplantation to the left ventricular (LV) anterolateral surface was conducted by Yoshikawa et al. [121]. Treatment with scaffold-free cell sheet-based therapies showed improved LV functions as the volume of LV decreased and the ejection fraction was enhanced. At the area of transplantation, thickening of the systolic wall was observed with no major cardiac adverse events.

The encouraging results acquired from earlier animal studies established the feasibility of conducting a human clinical trial for PDL cell sheet-based therapy to treat periodontitis. Feng et al. conducted a preclinical study and treated three periodontitis patients by transplantation of autologous PDL-derived stem cell (PDLSC) sheets [122]. No signs of adverse effects caused by PDLSC sheet transplantation were detected, and the study showed a significant enhancement of periodontal disease, which was evident by PDLSC sheet-associated cementum-like and PDL-like tissue formation in vivo eight weeks post-transplantation. In addition, CSE is also applicable to the regeneration of periodontal intrabony defects. According to reports by Chen et al., patients who were treated with autologous PDLSC sheets in combination with bovine-derived bone mineral materials showed a significant height increase and bone replacement in the alveolar bone over time, with no clinical safety issues [123]. Recently, Iwata et al. isolated autologous PDLSCs from patients with periodontitis and three-layered PDLSC sheets were fabricated using PNIPAAm-grafted TCPS [82]. In this single-arm and single-institute phase I clinical study, three-layered PDLSC sheets were transplanted into denuded root surface, and the treatment efficacy was assessed during a 12-month follow-up. The results indicated that there were no clinical safety issues, and patients treated with the PDLSC sheets showed a substantial increase in their alveolar bone height.

Taken together, the results from these preclinical/clinical trials have demonstrated efficient and functional recovery in patients who were treated with autologous cell sheets. As cell sheet implants are reported to offer better functional recovery than needle-injection methods, it is efficient in cases where needle puncture can cause severe drawbacks. Although other hurdles including cell source, cell viability upon cryopreservation, cell engraftment, and appropriate measure for graft rejection must be overcome or optimized, these promising results warrant further clinical follow-up studies to validate the therapeutic efficacy and safety of temperature-responsive CSE technologies. The different approaches in cell sheet-based technology described in this review are summarized in Table 1.

## 5. Conclusions

CSE uses temperature-responsive polymers—such as PNIPAAm and MC—to form unique, scaffold-free, and functionally engraftable cell sheets comprising a layer of cells maintaining critical intercellular junctions, cell-deposited extracellular matrix, and other important cell surface proteins that can be detached by changing the surface temperature. Dense or thick cell sheets can be designed for use in clinical applications alone or stacked on top of one another to form multi-layered constructs for specific tissue regeneration. CSE has been demonstrated to have high therapeutic potential in the treatment of various degenerative conditions. Animal studies, preclinical, and clinical studies have demonstrated the regenerative capacity of CSE-based approaches. Despite the promising results reported thus far, further studies are necessary for translating cell sheet-based therapy from bench to the bedside.

## Figures and Tables

**Table 1:** Various applications of cell sheet-based technology for treating bone, periodontal, skin, and vascularized muscles.

Applications	Methods for Cell Sheet Formation	Summarized Results	Refs.
**Bone**	Physical detachment	In vivo bone formation by transplanting bone marrow cell sheets	[68]
**Bone**	Poly(*N*-isopropylacrylamide)	New bone formation at day seven post-transplantation upon transplantation of rat bone marrow stromal cell sheet into subcutaneous site.	[69]
**Bone**	Poly(*N*-isopropyl acryl amide-*co*-methacrylic acid)	In vitro osteogenesis of human adipose-derived stem cell sheets validated by Alizarin red staining and qPCR.	[70]
**Bone**	Methylcellulose and poly(*N*-isopropylacrylamide)	In vitro osteogenesis of human adipose-derived stem cell sheets validated by ALP, Alizarin Red staining and qPCR	[71]
**PDL**	Poly(*N*-isopropylacrylamide)	Validation of safety and efficacy of autologous PDL-derived cell sheets	[82]
**PDL**	Poly(*N*-isopropylacrylamide)	Regeneration of PDL tissues in a rat mesial dehiscence model	[83]
**PDL**	Poly(*N*-isopropylacrylamide)	Newly formed cementum and well-oriented PDL fibers by PDL cell sheet transplantation	[84]
**PDL**	Poly(*N*-isopropylacrylamide)	New bone and cementum-like tissue formation in a canine periodontal defect model	[79]
**Skin**	Poly(*N*-isopropylacrylamide)	In vivo engraftment and skin regeneration by multiple stacks of hASC-derived 3D cell sheets in a full-thickness wound model	[93]
**Skin**	Poly(*N*-isopropylacrylamide)	Newly formed collagen matrix and blood vessel structures in a full-thickness wound model using hASC-derived cell sheets	[94]
**Skin**	Poly(*N*-isopropylacrylamide)	Accelerated wound closure and dermis tissue formation by rat ASC cell sheets	[96]
**Cardiac Muscle**	Poly(*N*-isopropylacrylamide)	Enhanced cardiac function by transplanted pre-vascularized cardiac cell sheets in a rat myocardial infarction model	[102]
**Cardiac Muscle**	Poly(*N*-isopropylacrylamide)	Recovery of the endocardium and capillary density by cardiac stem cell sheet and endothelial progenitors	[104]
**Cardiac Muscle**	Physical detachment	Significant functional recovery of the ischemic epicardium by SVF-derived cell sheets	[106]
**Smooth Muscle**	Poly(*N*-isopropylacrylamide) and polycaprolactone	Engineered uniaxially aligned and tubular blood vessel structure by smooth muscle cell sheets	[107]
**Skeletal Muscle**	Poly(*N*-isopropylacrylamide)	Uniaxially aligned skeletal muscle cell tissue formation by skeletal muscle cell sheets	[107,108]
**Skeletal Muscle**	Poly(*N*-isopropylacrylamide)	Uniaxially aligned smooth muscle cell sheets induced to form blood vessel-like cellular orientation	[108]
**Skeletal Muscle**	Poly(*N*-isopropylacrylamide)	Well-controlled 3D aligned skeletal cell sheets having physical and biological successful anisotropy	[110]
**Cardiac Muscle**	Poly(*N*-isopropylacrylamide)	Well-organized microvascular formation and enhanced heart function by cell-dense multiple layered myocardial cell sheet grafts	[111]
**Cardiac Muscle**	Poly(*N*-isopropylacrylamide)	Enhanced blood supply and cell viability by hiPSC-derived cell sheets	[112]
**Cardiac Muscle**	Methylcellulose	Improved LV functions by rat BM-MSC sheets in a rat myocardial infarction model	[113]
**Cardiac Muscle**	Methylcellulose	Improved LV functions by human AFSC-derived cell sheets in a rat myocardial infarction model	[114]
**Clinical trial: cardiac muscle**	Poly(*N*-isopropylacrylamide)	Improved cardiac functions by autologous myoblast cell sheets transplantation	[119]
**Clinical trial: cardiac muscle**	Poly(*N*-isopropylacrylamide)	Improved clinical condition without any arrhythmia and a left ventricular assist system	[120]
**Clinical trial: cardiac muscle**	Poly(*N*-isopropylacrylamide)	Improved LV functions by autologous cell sheets transplantation	[121]
**Clinical trial: periodontal ligament**	Poly(*N*-isopropylacrylamide)	Improved periodontitis symptoms by autologous PDL-derived stem cell sheets	[122]
**Clinical trial: periodontal ligament**	Poly(*N*-isopropylacrylamide)	Improved periodontitis symptoms by autologous PDL-derived stem cell sheets	[123]

## Abbreviations

hASC	Human adipose-derived stem cells
β-TCP	β-tricalciumphosphate
CSE	Cell sheet engineering
LCST	Lower critical solution temperature
LV	Left ventricular
MI	Myocardial infarction
PDL	Periodontal ligament
PSC	Pluripotent stem cells
SVF	Stromal vascular fraction
TCPS	Tissue culture polystyrene
UCST	Upper critical solution temperature
VEGF	Vascular endothelial growth factor
AVL	Arteriovenous loop
VPT	Volume phase transition
hESCs	Human embryonic stem cells
hMSCs	Human mesenchymal stem cells
hBM-MSCs	Human bone marrow-derived mesenchymal stem cells
hAFSCs	Human amniotic fluid stem cells
PNIPAAm	Poly(*N*-isopropylacrylamide)
MEO_2_MA	2-(2-methoxyethoxy) ethyl methacrylate
OEGMA	Oligo(ethylene glycol) methacrylate
hUCBMSCs	Human umbilical cord blood mesenchymal stem cells
CTX	Cardiotoxin
ECM	Extracellular matrix
PVME	Poly(vinyl methyl ether)
PVMEMA	Poly(vinyl methyl ether and maleic acid)
PGA	Poly(glycolic acid)
TA	Tibialis anterior
ASCs	Adipose-derived stem cells
PDMS	Polydimethylsiloxane
MC	Methylcellulose
PDEA	Poly(*N*,*N*-diethylacrylamide)
PEG	Poly(ethylene glycol)
PVA	Poly(vinyl alcohol)
PAA	Poly(acrylic acid)
PHEMA	Poly(2-hydroxyethyl methacrylate)
PLA	Poly(lactic acid)
PCL	Poly(caprolactone)
PNVCL	Poly(*N*-vinylcaprolactam)
PMAA	Poly(methacrylic acid)
PEO	Poly(ethylene oxide)
*n-*BMA	*n-*butyl methacrylate
PPO	Poly(propylene oxide)
PEA	Poly(ethyl acrylate)
p(NIPAAm-*co*-MMA)	Poly(*N*-isopropyl acryl amide-*co*-methacrylic acid)
SE	Secretome of ASCs
VC	Vitamin C
VEGF	Vascular endothelial growth factor
HGF	Hepatocyte growth factor
LV	Left ventricular
PDLSCs	PDL-derived stem cells
